# RIPK3 Facilitates Host Resistance to Oral Toxoplasma gondii Infection

**DOI:** 10.1128/IAI.00021-21

**Published:** 2021-04-16

**Authors:** Patrick W. Cervantes, Bruno Martorelli Di Genova, Billy Joel Erazo Flores, Laura J. Knoll

**Affiliations:** a Department of Medical Microbiology and Immunology, University of Wisconsin-Madison, Madison, Wisconsin, USA; UC Davis School of Veterinary Medicine

**Keywords:** *Toxoplasma gondii*, toxoplasmosis, ZBP1, RIPK3, cell death, necroptosis, pyroptosis

## Abstract

Toxoplasma gondii infection activates pattern recognition receptor (PRR) pathways that drive innate inflammatory responses to control infection. Necroptosis is a proinflammatory cell death pathway apart from the innate immune response that has evolved to control pathogenic infection. In this study, we further defined the role of Z-DNA binding protein 1 (ZBP1) as a PRR and assessed its contribution to necroptosis as a host protection mechanism to T. gondii infection. We found that ZBP1 does not induce proinflammatory necroptosis cell death, and ZBP1 null mice have reduced survival after oral T. gondii infection. In contrast, mice deleted in receptor-interacting serine/threonine-protein kinase 3 (RIPK3^−/−^), a central mediator of necroptosis, have significantly improved survival after oral T. gondii infection without a reduction in parasite burden. The physiological consequences of RIPK3 activity did not show any differences in intestine villus immunopathology, but RIPK3^−/−^ mice showed higher immune cell infiltration and edema in the lamina propria. The contribution of necroptosis to host survival was clarified with mixed-lineage kinase domain-like pseudokinase null (MLKL^−/−^) mice. We found MLKL^−/−^ mice succumbed to oral T. gondii infection the same as wild-type mice, indicating necroptosis-independent RIPK3 activity impacts host survival. These results provide new insights on the impacts of proinflammatory cell death pathways as a mechanism of host defense to oral T. gondii infection.

## Abstract (precis)

Toxoplasma gondii infection activates pattern recognition receptor (PRR) pathways that drive innate inflammatory responses to control infection. Necroptosis is a proinflammatory cell death pathway apart from the innate immune response that has evolved to control pathogenic infection.

## INTRODUCTION

Toxoplasma gondii is one of the most widespread parasitic infections in the world and is acquired by nearly 30% of the human population (1). The natural route of infection occurs by consuming food or water contaminated with tissue cysts from the asexual cycle or oocysts from the sexual cycle. Asexual cysts contain the bradyzoite stage, which differentiates into the tachyzoite stage after ingestion and invasion of the intestinal tract. Tachyzoites disseminate from the intestine throughout the body during acute infection. Host immune pressure causes tachyzoite parasites to differentiate into bradyzoite cysts that reside in the brain and muscle tissue. This stage constitutes a latent, chronic infection. Pathogen recognition and a balanced inflammatory immune response are necessary to survive an acute infection.

T. gondii elicits a strong T helper type 1 (Th1) response, characterized by interleukin-12 (IL-12)-induced interferon gamma (IFN-γ), which in turn orchestrates cell-mediated immunity important for intracellular pathogen defense (2). Deficiencies in this pathway lead to uncontrolled parasite replication and host death (1, 3, 4), highlighted in mouse studies where IL-12, IFN-γ, toll-like receptor (TLR), and myeloid differentiation primary response 88 (MyD88) responses are perturbed (5–10). These signaling pathways, cytokines, and their downstream effectors are elevated throughout long-term chronic infection and must be maintained to prevent cyst reactivation and fatal encephalitis (11, 12).

In contrast to a weak Th1 response, a hyperactive and unregulated Th1 response results in severe tissue damage and host death (13, 14). Immune regulation by IL-10 is vital to host survival, because IL-10 null mice succumb to oral T. gondii infection due to uncontrolled host immune response and not uncontrolled parasite replication (13, 14). Early studies in susceptible C57BL/6J mice used high-dose oral T. gondii infections and showed mortality is caused by an excessive immune response that is accompanied by severe intestinal immunopathology and tissue necrosis (15–17). This fatal pathology is mediated by the uncontrolled expression of IFN-γ, tumor necrosis factor (TNF), and nitric oxide (15, 16). Therefore, a balanced innate immune response is essential for a host to survive acute T. gondii infection.

The innate immune system employs pattern recognition receptors (PRRs) to identify pathogens. Common PRRs are TLRs, nucleotide oligomerization domain-like receptors (NLR), and DNA/RNA sensors, like Z-DNA binding protein-1 (ZBP1). These PRRs induce programmed cell death pathways as a mechanism for host defense to infectious diseases (18, 19). Programmed cell death pathways include apoptosis, necroptosis, and pyroptosis. Apoptosis is a cell death pathway regarded as noninflammatory, whereas necroptosis and pyroptosis are lytic forms of cell death that release highly proinflammatory damage-associated molecular compounds (20, 21). Central to necroptosis is receptor-interacting serine/threonine-protein kinase 3 (RIPK3) and its downstream substrate, mixed lineage kinase domain-like pseudokinase (MLKL). Similarly, pyroptosis depends on caspase-1 activation that is mediated by NLR inflammasome components. The processes of necroptosis and pyroptosis ultimately induce cell membrane pores and lytic cell death that contribute to inflammatory immune responses, adaptive immunity, and host defense against pathogenic infection.

Our laboratory previously found *Zbp1* transcripts are highly expressed in the brains of mice chronically infected with T. gondii (11, 22). We also found ZBP1 assists in host control of T. gondii infection *in vitro* and *in vivo* (23). Recently, IFN-γ was shown to strongly induce ZBP1 to complex with RIPK3 to mediate inflammation and necroptosis as a host defense mechanism (24–27). Here, we tested the hypothesis that ZBP1-dependent necroptosis is protective for T. gondii infection.

## RESULTS

### ZBP1^−/−^ and RIPK3^−/−^ mice show divergent phenotypes to necroptosis and host survival.

The ZBP1 knockout mice (ZBP1^−/−^) used in Pittman et al. (23) appear to have a mixed genetic background, complicating comparisons to wild-type (WT) mice (28). To rectify this, we created a new ZBP1^−/−^ mouse in the C57BL/6 background using CRISPR-Cas9 technology to remove the entire *Zbp1* genomic locus, including the promoter and all mRNA splice variants (see Fig. S1 in the supplemental material). We then evaluated the role of ZBP1 in host protection during T. gondii infection.

**FIG 1:**
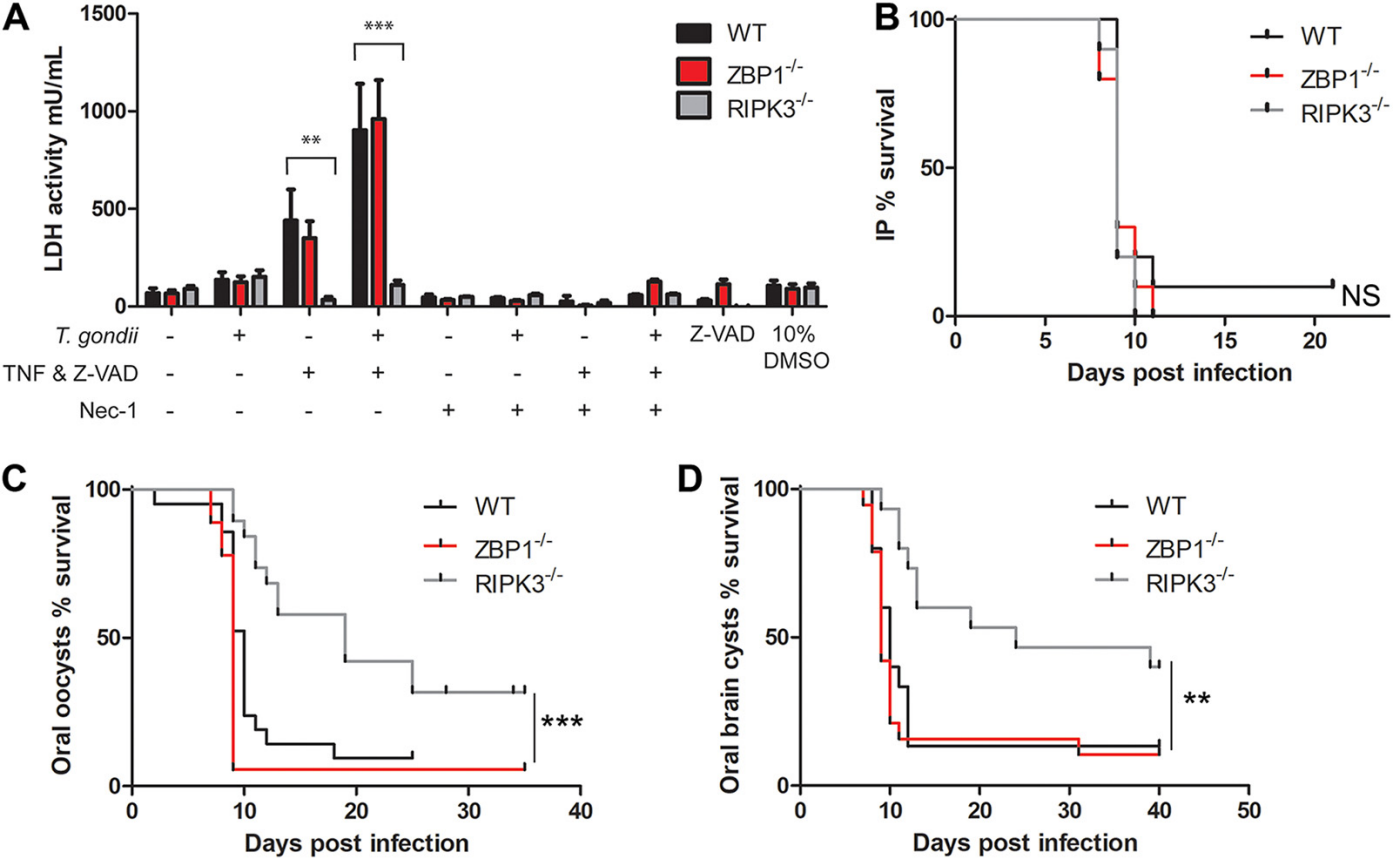
ZBP1 and RIPK3 show divergent phenotypes to necroptosis and host survival. (A) Necroptosis was measured by LDH release in BMDM. Cells were seeded at 5 × 10^4^ cells/well in triplicate in a 96-well plate and infected with 2.5 × 10^5^ parasites/well or left uninfected for 3 h. Stimulated cells received 50 ng/ml TNF and 40 μM Z-VAD-FMK, and unstimulated cells received fresh media. After 24 h, the medium supernatant was used to measure LDH release by absorption. Necrostatin-1 was added upon stimulation at 50 μM as a negative control for necroptosis. These results are from 2 independent experiments. A 2-way analysis of variance (ANOVA) with Bonferroni posttest was used for statistical analysis in LDH release. **, *P* < 0.01; ***, *P* < 0.001. (B) Host survival from i.p. inoculation with T. gondii tachyzoites. Female (WT, *n* = 10; ZBP1^−/−^, *n* = 10; RIPK3^−/−^, *n* = 10) mice were i.p. infected with 1 × 10^4^ tachyzoites/mouse. These data combine 2 independent experiments. Significance was determined by a log-rank (Mantel-Cox) test, and the *P* value between genotypes was not significant (NS). (C) Host survival from an oral infection with oocysts. Male (WT, *n* = 17; ZBP1^−/−^, *n* = 12; RIPK3^−/−^, *n* = 15) and female (WT, *n* = 9; ZBP1^−/−^, *n* = 6; RIPK3^−/−^, *n* = 8) mice were orally infected by gavage with 6 × 10^3^ mCherry oocysts/mouse in 3 independent experiments. Significance was determined by a log-rank (Mantel-Cox) test. The *P* value was 0.001 (***) for the WT compared to RIPK3^−/−^ mice, and the *P* value was not significant between WT and ZBP1^−/−^ mice. (D) Host survival from an oral infection with brain tissue cysts. Female (WT, *n* = 15; ZBP1^−/−^, *n* = 17; RIPK3^−/−^, *n* = 15) mice were orally infected by gavage with 4 × 10^3^ brain tissue cysts in 3 independent experiments. Significance was determined by a log-rank (Mantel-Cox) test. The *P* value was 0.003 (**) between WT and RIPK3^−/−^ mice, and the *P* value was not significant between WT and ZBP1^−/−^ mice.

Necroptosis is a proinflammatory cell death pathway where cells become permeable and release damage-associated molecular compounds that enhance inflammatory responses (20). Because ZBP1 has been shown to drive RIPK3-dependent necroptosis (29), we tested whether ZBP1 could activate this pathway during T. gondii infection. We included RIPK3 null (RIPK3^−/−^) mice as a negative control for necroptosis, because RIPK3 kinase and scaffold domains are essential for necroptosis (30, 31). Necroptosis was measured in WT, ZBP1^−/−^, and RIPK3^−/−^ bone marrow-derived macrophages (BMDM) by lactate dehydrogenase (LDH) release. LDH release was reduced in all genotypes treated with necrostatin-1, a necroptosis inhibitor (Fig. 1A). Infection with T. gondii alone caused little LDH release in each BMDM genetic background, but stimulation with TNF and Z-VAD-FMK (a pan-caspase inhibitor that drives necroptosis) caused a similar increase in LDH release in WT and ZBP1^−/−^ BMDM. The LDH release in WT and ZBP1^−/−^ BMDM was amplified when stimulation with TNF and Z-VAD-FMK was combined with T. gondii infection (Fig. 1A). In contrast, RIPK3^−/−^ BMDM showed no LDH release above background under any condition. This outcome indicates that ZBP1 does not promote cell permeability, a process apart from necroptosis, while RIPK3 influences cell membrane integrity during T. gondii infection.

Proinflammatory cell death pathways, like necroptosis, have evolved to protect the host from pathogenic infections (18). An acute host survival challenge is a simple assay to determine a gene’s contribution to host protection during infection. We established host protection against T. gondii by intraperitoneal (i.p.) and oral infection. Similar to previous observations (20), there was no significant difference between WT and ZBP1^−/−^ mouse survival after i.p. inoculation (Fig. 1B), but the ZBP1^−/−^ mice were more susceptible to oral T. gondii challenges with oocysts and brain tissue cysts (Fig. 1C and D and Fig. S2 to S4). Along with ZBP1^−/−^ mice, the RIPK3^−/−^ mice also showed no difference in survival after i.p. inoculation with T. gondii tachyzoites (Fig. 1B). However, unlike ZBP1^−/−^ mice, the RIPK3^−/−^ mice had significantly improved survival after oral infection with both T. gondii oocysts and brain tissue cysts (Fig. 1C and D). These results suggest that while ZBP1 and RIPK3 both play roles during oral T. gondii infection, they are likely working in different pathways.

### RIPK3^−/−^ mice do not have lower parasite burdens.

**FIG 2:**
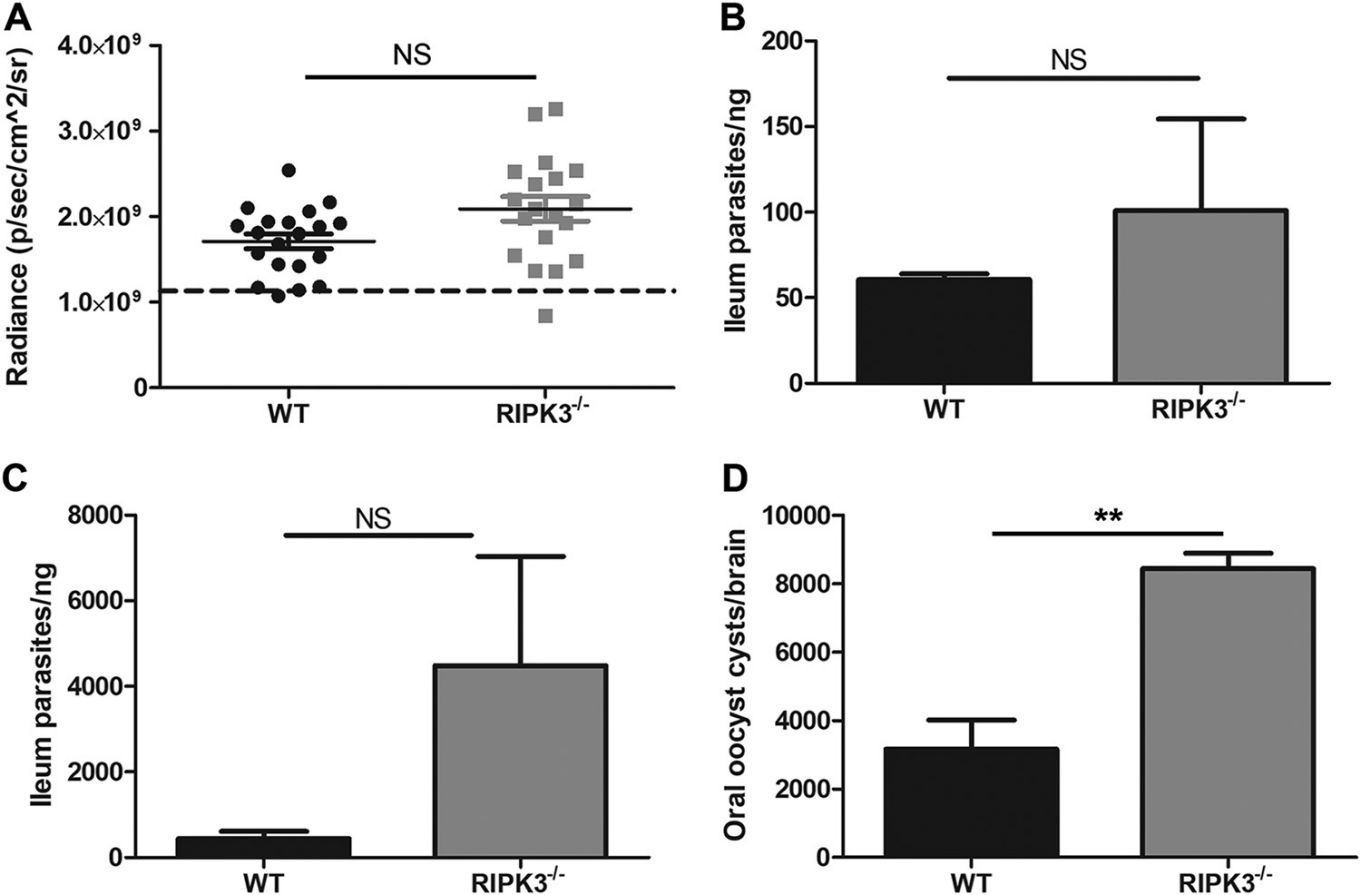
RIPK3^−/−^ mice do not have lower parasite burdens. (A) Intestine parasite burden determined by mCherry fluorescence with IVIS. Male (WT, *n* = 10; RIPK3^−/−^, *n* = 9) and female (WT, *n* = 10; RIPK3^−/−^, *n* = 10) mice were gavage fed 1 × 10^4^ mCherry oocysts, their intestines were removed, and mCherry fluorescence was measured (excitation, 587 nm; emission, 610 nm) by IVIS at 7 days postinfection. The dotted line indicates the average background mCherry fluorescence of uninfected male (WT, *n* = 2; RIPK3^−/−^, *n* = 2) and female (WT, *n* = 4; RIPK3^−/−^, *n* = 3) mouse intestines. (B) Parasite burden measured by qPCR in intestines with parasite-specific SAG1 primers at 7 days postinfection (dpi). Total gDNA was extracted from 1 cm ileum, next to the cecum, from female mice (WT, *n* = 3; RIPK3^−/−^, *n* = 3) orally infected with 3 × 10^3^ mCherry oocysts. A standard curve was generated from a known concentration of tachyzoite parasites to calculate the burden. The *P* value was not significant (NS). (C) Parasite burden measured by qPCR in intestines with parasite-specific B1 primers at 7 dpi. Total gDNA was extracted from 1 cm ileum, next to the cecum, from female mice (WT, *n* = 3; RIPK3^−/−^, *n* = 3) orally infected with 8 × 10^4^ mCherry oocysts. A standard curve was generated from a known concentration of tachyzoite parasites to calculate the burden. The *P* value was not significant (NS). (D) Brain cyst burden in male (WT, *n* = 5; RIPK3^−/−^, *n* = 3) mice 28 days after oral infection with 3 × 10^3^ mCherry oocysts.

We sought to understand the role of RIPK3 in host defense responses given the striking survival phenotype for oral infection. Genetic evidence supports that RIPK3-dependent necroptosis clears viral, bacterial, and parasitic infections that can affect host susceptibility (24, 25, 31–35). Therefore, we hypothesized that RIPK3^−/−^ mice would have a lower parasite burden than WT mice. We first performed a qualitative assessment of the T. gondii burden at the peak acute infection by measuring mCherry parasite fluorescence with an *in vivo* imaging system (IVIS). The mCherry signal was not reduced in RIPK3^−/−^ intestines compared to WT intestines (Fig. 2A). We quantified the parasite burden in the intestine and liver by quantitative PCR (qPCR) at 7 and 9 days after a sublethal mCherry oocyst dose (Fig. 2B and Fig. S5). While there was a consistently increased parasite burden in infected RIPK3^−/−^ mice, the difference was not significant. We then quantified the parasite burden in the ileum after a lethal mCherry oocyst dose by qPCR at 7 days postinfection (Fig. 2C). We again saw an increased parasite burden in infected RIPK3^−/−^ mice, but due to the variability of the infection in RIPK3^−/−^ mice, this difference was not significant. We also quantified the brain cyst burden in WT and RIPK3^−/−^ mice 28 days after a sublethal mCherry oocyst dose. We saw a significant increase in the number of brain cysts in RIPK3^−/−^ compared to WT mice (Fig. 2D). Overall, these results show that infected RIPK3^−/−^ mice do not have a lower parasite burden than WT mice and may even have a slightly higher parasite burden that continues into chronic infection.

### T. gondii does not survive and replicate faster in RIPK3^−/−^ macrophages.

**FIG 3:**
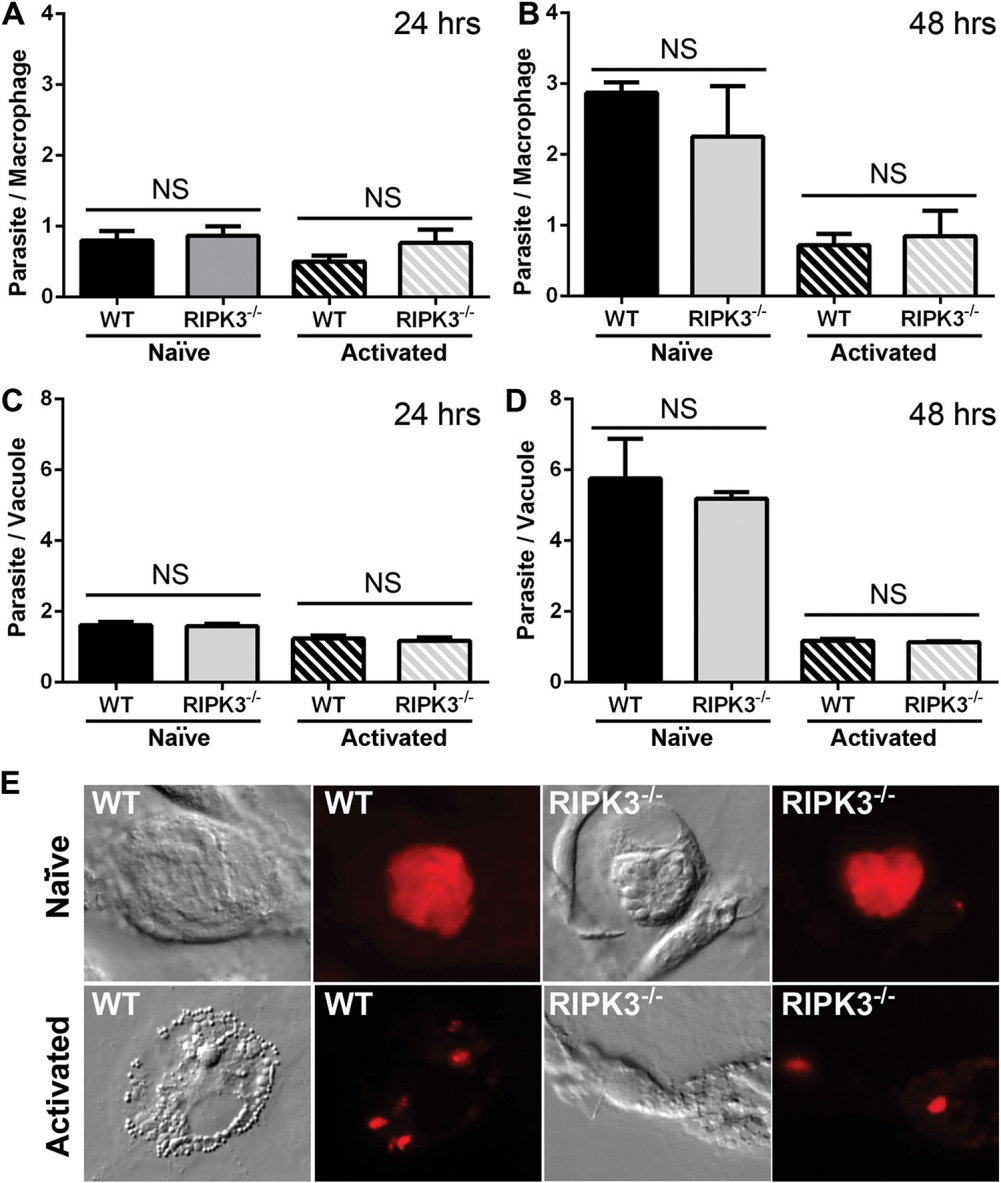
T. gondii does not replicate faster in RIPK3^−/−^ macrophages. WT and RIPK3^−/−^ BMDM were infected with mCherry parasites. At 3 h postinfection, cells were either left naive or stimulated with LPS and IFN-γ. Total parasites per macrophage were counted at 24 h (WT, *n* = 6; RIPK3^−/−^, *n* = 6) (A) and 48 h (WT, *n* = 6; RIPK3^−/−^, *n* = 6) (B) postinfection in at least 170 macrophages. A 2-tailed independent Student’s *t* test was used to calculate significance. All pairwise WT-to-RIPK^−/−^ comparisons were not significant (NS). For these same slides, the total parasites per vacuole were counted at 24 h (WT, *n* = 6; RIPK3^−/−^, *n* = 6) (C) and 48 h (WT, *n* = 6; RIPK3^−/−^, *n* = 6) (D) postinfection in at least 260 vacuoles. All pairwise WT-to-RIPK^−/−^ comparisons were not significant (NS). WT naïve versus activated at 24 h postinfection, *P* < 0.01 (**); RIPK3 naïve versus activated at 24 h postinfection, *, *P* < 0.05 (*); WT naïve versus activated at 48 h postinfection, *P* < 0.05 (*); RIPK3 naïve versus activated at 48 h postinfection (***), *P* < 0.001. (E) Example photographs of infected vacuoles at 48 h postinfection. Macrophages were imaged with differential contrast imaging, and parasites were imaged with the Texas Red filter to show mCherry fluorescence.

Because of the slight increase in parasite burden in infected RIPK3^−/−^ mice, we examined the total number of parasites per macrophage as well as the replication rate of T. gondii in WT and RIPK3^−/−^ BMDM. Macrophages were infected with mCherry tachyzoites for 3 h and then either left naive or stimulated with LPS and IFN-γ. After 24 and 48 h, the total number of parasites per macrophage and the number of parasites in each vacuole were counted on blinded slides. While T. gondii replicated significantly faster in naive than activated macrophages, no significant difference was seen between WT and RIPK3^−/−^ macrophages, either naive or activated, in the number of parasites per macrophage or the number of parasites in each vacuole (Fig. 3). These results indicate that the difference in parasite burden during animal infection is not due to increased survival or replication rate of T. gondii in RIPK3^−/−^ macrophages.

### WT and RIPK3^−/−^ mice do not have differences in intestinal villus pathology.

**FIG 4:**
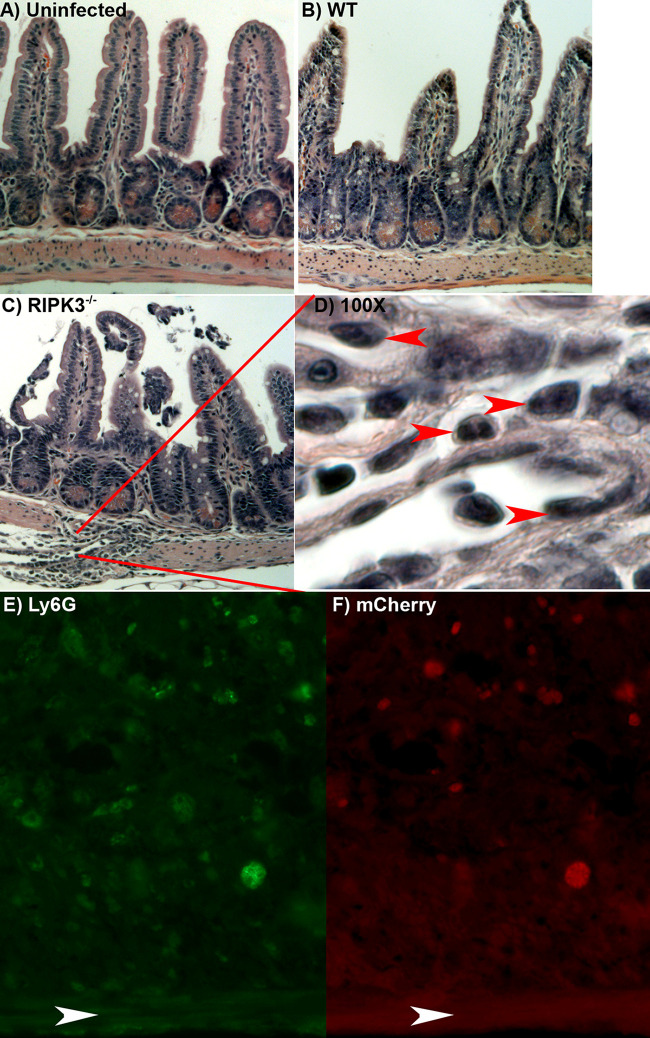
Intestinal histopathology shows similar villus damage in WT and RIPK3^−/−^ mice. Wild-type (WT) female mice were left uninfected (A) or infected with 600 mCherry oocysts by oral gavage (B), or RIPK3^−/−^ mice were infected with 600 mCherry oocysts by oral gavage (C). All mice were sacrificed at day 7 postinfection, and the ileum (distal 1/3 of the small intestine) was washed with PBS and fixed in 10% buffered formalin in a Swiss roll, sectioned, and then stained with H&E and imaged with the 10× objective on a Zeiss light microscope. The red lines indicate the area of panel C that was imaged with the 100× objective for panel D. The red arrowheads indicate inflammatory cells that are likely neutrophils. (E and F) The white arrowheads indicate the muscularis at the bottom of the photo. The lamina propria of the ileum from infected RIPK3^−/−^ mice were stained for Ly6G (E, green) and imaged for mCherry expressing parasites (F, red) using the 40× objective.

**FIG 5:**
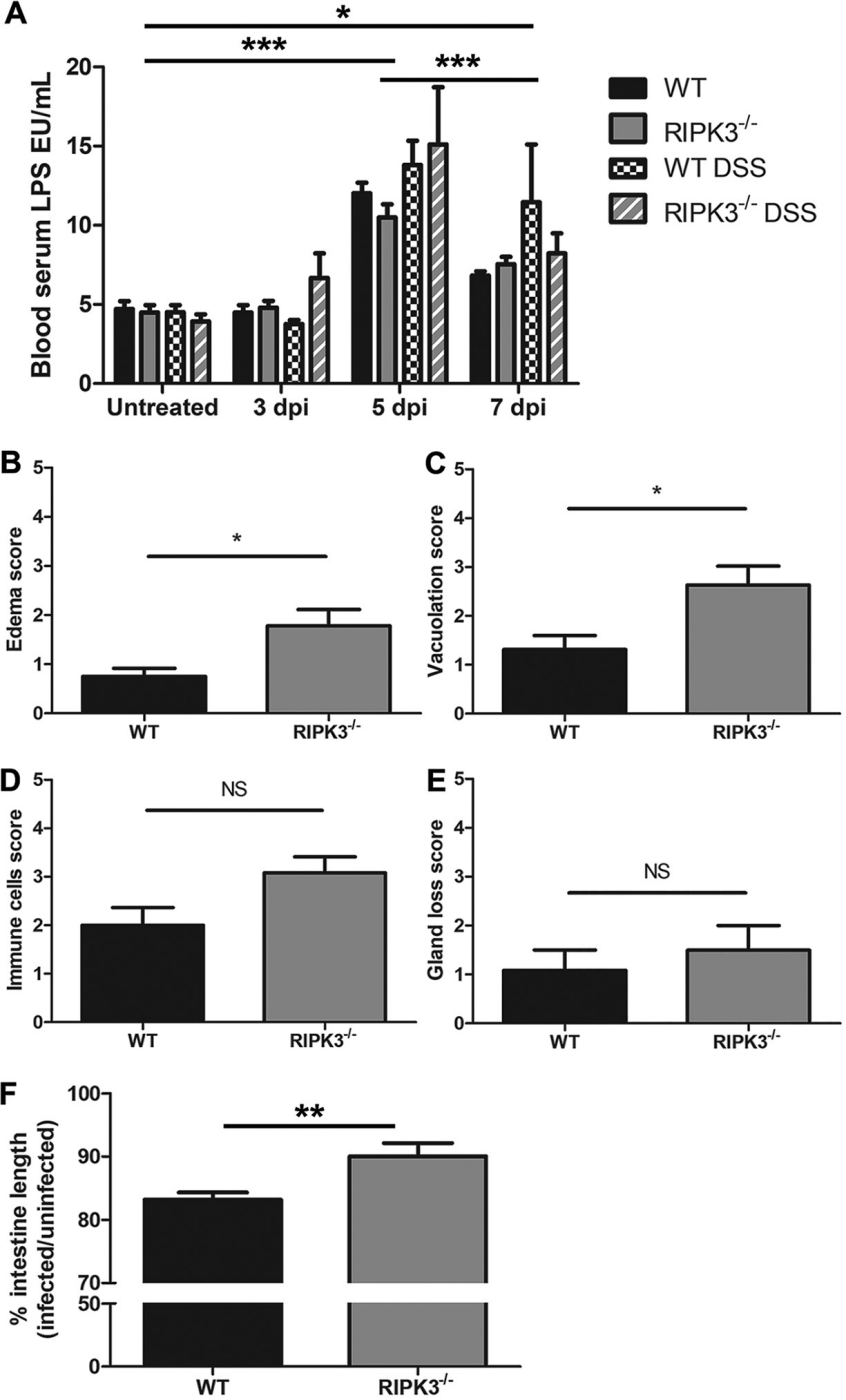
RIPK3 does not affect intestinal permeability but lamia propria pathology after oral T. gondii infection. (A) Intestine permeability by LPS concentration in blood serum. Male (WT, *n* = 4; RIPK3^−/−^, *n* = 3) and female (WT, *n* = 5; RIPK3^−/−^, *n* = 5) mice were orally infected with 1 × 10^4^ mCherry oocysts by gavage. As a positive control, male (WT, *n* = 4; RIPK3^−/−^, *n* = 5) and female (WT, *n* = 4; RIPK3^−/−^, *n* = 3) mice were treated with 3% DSS in drinking water. Paired blood serum was collected at days 0 (uninfected), 3, 5, and 7 after oral infection. Statistical significance was calculated by 2-way ANOVA with Bonferroni posttest. ***, *P* < 0.001 for uninfected compared to 5 dpi and 5 dpi compared to 7 dpi. *, *P* < 0.05 for uninfected compared to 7 dpi. There was no significant difference within each group. (B to E) Intestine pathology scores by H&E in ileum Swiss rolls. Female mice (WT, *n* = 6; RIPK3^−/−^, *n* = 6) were infected with 600 mCherry oocysts by oral gavage and sacrificed at day 7 postinfection. The ileum (distal 1/3 of the small intestine) was washed with PBS and fixed in 10% buffered formalin in a Swiss roll. Uninfected female mouse (WT, *n* = 3; RIPK3^−/−^, *n* = 4) ileum samples were processed the same as controls. The slides were blinded and scored from 0 to 5 (0, equivalent to control; 5, severe) for edema (*, *P* < 0.05) (B), smooth muscle vacuolation (*, *P* < 0.05) (C), immune cell infiltration (not significant [NS]) (D), and gland loss (not significant [NS]) (E). (F) Intestine length was measured in male (WT, *n* = 6; RIPK3^−/−^, *n* = 6) and female (WT, *n* = 6; RIPK3^−/−^, *n* = 6) mice at 7 days after oral infection by gavage with 1 × 10^4^ mCherry oocysts. A 2-tailed independent Student’s *t* test was used to calculate significance from 2 independent experiments. **, *P* < 0.01.

As RIPK3 is associated with mucosal immune pathology (36, 37) and oral T. gondii infection causes intestinal inflammation (38–41), we assessed intestinal villus immunopathology. At day 7 after oral T. gondii infection, hematoxylin and eosin (H&E)-stained ileum Swiss rolls were blinded and assessed for villus integrity. While we saw signs of villus damage due to oral T. gondii infection, there were no differences in villus damage between WT and RIPK3^−/−^ mice (Fig. 4). We further examined villus integrity by measuring intestine permeability, because it was previously seen that oral T. gondii infection causes lipopolysaccharide (LPS) from the bacterial microbiome as well as gavage-fed fluorescein isothiocyanate (FITC)-dextran to enter the circulation (42). Using a dextran sulfate sodium (DSS)-induced colitis model as a positive control, we measured LPS in the blood at 3, 5, and 7 days after oral T. gondii infection. There were no significant differences in serum LPS (Fig. 5A) or gavage-fed FITC-dextran concentration in blood serum between WT and RIPK3^−/−^ mice (Fig. S6). These results suggest that intestine permeability induced by oral T. gondii infection is equivalent in both strains, and the increased survival seen in RIPK3^−/−^ mice after oral T. gondii infection is not due to decreased intestinal villus pathology.

### RIPK3^−/−^ mice have more inflammation in the lamina propria.

After oral infection, T. gondii parasites can be found largely in the lamina propria of the ileum (43–45). We noticed that large patches of immune cells tended to be more common in the lamina propria of RIPK3^−/−^ mice after oral T. gondii infection (Fig. 4C). Magnification of these areas showed many immune cells, including multilobed nucleated cells, which are indicative of neutrophils (Fig. 4D). Immunohistochemistry with the lymphocyte antigen 6 complex (Ly6G), which is a marker of monocytes, granulocytes, and neutrophils, showed many Ly6G-positive immune cells in the lamina propria of RIPK3^−/−^ mice. These Ly6G-positive cells were often, but not always, localized near T. gondii-infected cells (Fig. 4E and F).

We also submitted the H&E-stained ileum Swiss rolls to the UW-Madison Comparative Pathology Laboratory for analysis. They scored gland loss, immune cell infiltration, edema in the lamina propria, and vacuolation of the muscularis mucosa (Fig. 5B to E). There was a trend for the RIPK3^−/−^ mice to have increased inflammatory measures compared to WT mice, with scores for edema and vacuolation reaching statistical significance. We then measured the length of the entire infected small intestine, because intestine shortening is associated with tissue damage and pathology from oral T. gondii infections (38–41). The difference in intestine length was significant (Fig. 5F), as WT mice lost 17% intestine length compared to a 10% loss in RIPK3^−/−^ mice. The longer RIPK3^−/−^ intestine length could be due to swelling from increased edema, immune cell infiltration, and muscularis vacuolation, or it could be due to decreased immunopathology.

### RIPK3^−/−^ mice have higher IFN-γ and lower IL-10 levels.

**FIG 6:**
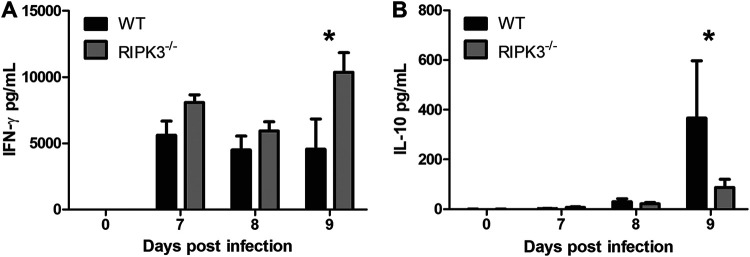
RIPK3 activity affects IFN-γ and IL-10 serum cytokine levels. Blood serum cytokines in female (WT, *n* = 14; RIPK3^−/−^, *n* = 15) mice gavage fed 6 × 10^3^ mCherry oocysts. Serum samples were collected at 7, 8, and 9 days postinfection in 3 independent experiments. IFN-γ (A) and IL-10 (B) levels were significantly different. A 2-way ANOVA with Bonferroni posttest was used to calculate significance. *, *P* < 0.05; all other pairwise comparisons were not significant.

As inflammatory cytokines are critical for the control of T. gondii infection, we compared serum cytokine levels between WT and RIPK3^−/−^ mice using the cytometric bead array mouse inflammation kit. There were no differences in monocyte chemoattractant protein-1 (MCP-1), IL-6, IL-12, and TNF systemic cytokine levels between WT and RIPK3^−/−^ mice at any of the time points examined (Fig. S7A to D). However, at day 9 after oral infection, RIPK3^−/−^ mice had significantly higher IFN-γ (Fig. 6A) and reduced IL-10 (Fig. 6B) levels. IFN-γ and IL-10 are both essential for mice to survive acute T. gondii infection, but IFN-γ is necessary to control parasitemia (9, 10) and IL-10 is necessary to control the host immune response (13, 14).

### MLKL^−/−^ and RIPK3^−/−^ mice show divergent host survival phenotypes.

**FIG 7:**
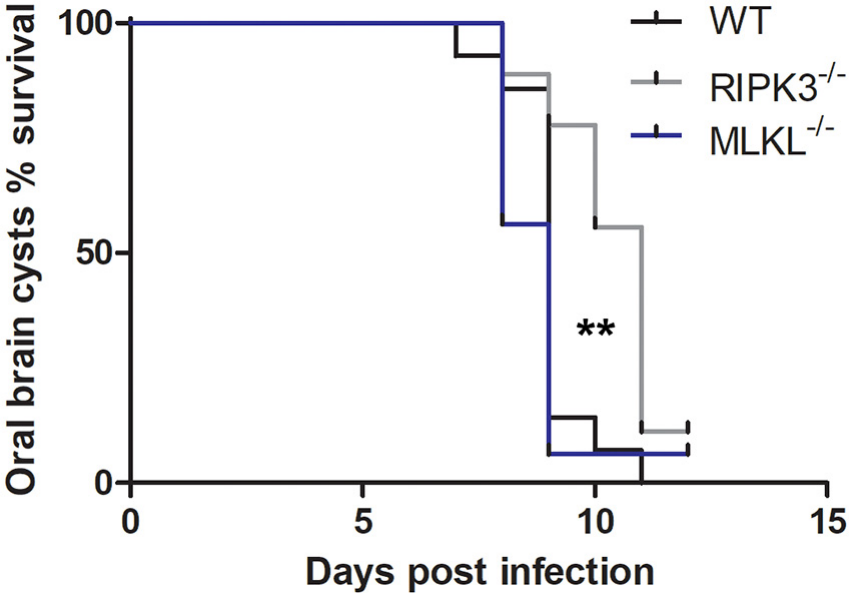
RIPK3-independent necroptosis activity influences host survival of oral infection. Female (WT, *n* = 25; RIPK3^−/−^, *n* = 16; MLKL^−/−^, *n* = 16) mice were orally infected with 4 × 10^3^ brain tissue cysts by gavage. A log-rank (Mantel-Cox) test was used to calculate the significance of each genotype. **, *P* < 0.01. The results are a compilation of 3 independent experiments.

Necroptosis-induced cell death and inflammation are dependent on RIPK3, but RIPK3 activity is not limited to necroptosis (46). The necroptosis executioner, MLKL, is the downstream substrate of RIPK3 that ultimately compromises cell membranes, fulfilling the necroptotic pathway (47). We specifically examined RIPK3-dependent necroptosis in host survival of oral T. gondii infection with mixed-lineage kinase domain-like pseudokinase null (MLKL^−/−^) mice. After oral infection, MLKL^−/−^ mice succumb to infection, similar to WT mice, whereas RIPK3^−/−^ mice showed improved survival (Fig. 7 and Fig. S8). These data provide evidence that RIPK3-independent necroptosis contributes to severe intestine pathology and host death.

## DISCUSSION

Innate immune activation and its role in host defense to pathogenic infections depend on PRR-driven inflammation. ZBP1 has recently come to light as a PRR involved in innate immunity and programmed cell death (29). Mechanisms of programmed cell death include apoptosis, necroptosis, and pyroptosis. T. gondii has evolved mechanisms to inhibit apoptosis to promote fitness (48). However, the host maintains cellular cross talk between each programmed cell death pathway to subvert apoptosis inhibition and guard against pathogenic infection via necroptosis and pyroptosis (18). ZBP1 was found to mediate the interferon-induced necroptosis pathway in response to viral infection (26, 27). ZBP1 as a PRR that induces immunity could fit within the realm of bacterial and parasitic infection (23, 49–51). The fact that IFN-γ and ZBP1 are highly expressed and maintained throughout T. gondii infection (11, 22) makes T. gondii a thought-provoking target to investigate the role of ZBP1 in nonviral infections. Our laboratory previously found ZBP1 was important for oral but not i.p. infection (23). Here, we sought to determine the potential role of ZBP1 in host-induced necroptosis in response to T. gondii infection by comparing ZBP1^−/−^ and RIPK3^−/−^ mice.

We tested if ZBP1 induced necroptosis by LDH release in BMDM after stimulation with TNF-α and Z-VAD-FMK and T. gondii infection. When the TNF-α receptor is activated and caspase-8 activity is inhibited by Z-VAD-FMK, RIPK3 phosphorylates MLKL to initiate necroptosis and cell permeability (52, 53). We found that the release of LDH was significantly reduced in RIPK3^−/−^ macrophages, indicating that T. gondii infection induces necroptosis in WT macrophages stimulated with TNF-α and Z-VAD-FMK. We also found ZBP1 to be independent of necroptosis after stimulation and T. gondii infection in BMDM (Fig. 1A). In response to oral T. gondii infection, ZBP1^−/−^ and RIPK3^−/−^ mice have opposing responses, with ZBP1^−/−^ mice being more susceptible (23) and RIPK3^−/−^ mice being more resistant (Fig. 1C and D). Studies using high-dose oral T. gondii infection have linked excessive Th1 immunity and intestinal proinflammatory cell death as the culprits in host mortality (15–17). The physiological role of RIPK3-dependent necroptosis can drive immunopathology and impair host fitness (30, 31, 36, 37). These findings support the fact that RIPK3^−/−^ mice have improved survival to oral T. gondii infection without a reduced parasite burden (Fig. 2). While there were no differences between WT and RIPK3^−/−^ mice in intestinal villus damage, there was a trend for the RIPK3^−/−^ mice to have increased inflammatory measures in the lamina propria (Fig. 4 and 5) as well as a reduction in IL-10 (Fig. 6). IL-10 is upregulated to control excessive Th1 cells and prevent immunopathology (54) and has previously been seen to be key in host survival after T. gondii infection (13, 14). All of these factors contradict a model where RIPK3^−/−^ mice survive oral T. gondii infection better than WT mice because of reduced immunopathology. RIPK3^−/−^ mice have reduced intestinal length loss after oral T. gondii infection compared to WT mice (Fig. 5F). This difference could be due to the WT mice having RIPK3-dependent activity causing more severe intestinal immunopathology, or, more likely, it could be that the small intestine in the RIPK3^−/−^ mice is more swollen and longer with the increased edema, immune cell infiltration, and muscularis vacuolation (Fig. 5B to D).

Gut bacteria drive an unregulated immune response that results in early host death, cellular necrosis, and severe tissue pathology in susceptible C57BL/6 WT mice in high-dose oral T. gondii infections (39, 55). The inflammatory response reduces antimicrobial compound secretion and compromises the epithelial barrier, which allows greater interaction with gut bacteria to promote Th1 immunity and pathogen control (42). Gnotobiotic- and antibiotic-treated mice are one of the few models that have also shown improved survival and higher parasite burdens after lethal oral T. gondii infections (39), similar to our RIPK3^−/−^ mice. Therefore, we examined intestine permeability by measuring bacterial LPS or gavage-fed FITC-dextran concentration in blood serum after oral T. gondii infection. There was no difference in LPS or FITC-dextran concentration in blood serum between WT and RIPK3^−/−^ mice (Fig. 5A; see also Fig. S6 in the supplemental material), which suggests intestine permeability and the degree of immune activation by gut bacteria are equivalent.

The biological function of RIPK3 appears to be important only for the natural route of T. gondii infection, as there is not a difference in susceptibility between WT and RIPK3^−/−^ mice after i.p. infections. This implicates the importance of the exposure route when studying host responses to pathogenic infections. The difference in RIPK3^−/−^ mouse survival between i.p. and oral infection likely contrast in pathogen recognition pathways that lead to immune stimulation. This result was evident in TLR-11 null (TLR-11^−/−^) mice that also have improved survival to oral but not i.p. T. gondii infection (56). TLR-11 in mice is a PRR that recognizes T. gondii profilin, which activates MyD88-dependent signaling pathways to induce IL-12 and IFN-γ, two critical cytokines for host survival of T. gondii infection (57). When TLR-11^−/−^ mice are challenged with T. gondii by i.p. infection, the host is unable to recognize the pathogen and mount an appropriate immune response, and, as a consequence, is overcome by uncontrolled parasite replication. In contrast, oral infection generates a TLR-11-independent protective immune response from gut bacteria that improves host survival. This study points toward an important role for gut bacteria to stimulate PRRs and immunity in the intestine to help fight pathogenic infections.

The role of RIPK3 is not limited to necroptosis. RIPK3 can activate pyroptosis as an alternative cell death mechanism that releases the proinflammatory effectors IL-1β and IL-18. Pyroptosis and its proinflammatory effectors are activated upon T. gondii infection and have been shown to control parasite burden in *in vitro* and *in vivo* oral infection models (58, 59). The proportion of RIPK3 activity in necroptosis or pyroptosis in host survival to oral T. gondii infection was clarified with MLKL^−/−^ mice. The observation that MLKL^−/−^ mice succumb to oral infection with WT mice indicated that necroptosis does not play a major role in the survival difference between WT and RIPK3^−/−^ mice. The degree of RIPK3^−/−^ protection on host survival of oral infection with brain cysts was reduced in the experiments for Fig. 7 compared to RIPK3^−/−^ mouse survival experiments in Fig. 1D. The variability in RIPK3^−/−^ survival is likely attributed to variance in the number of brain cysts used for oral infection. Because cyst wall staining and accurate counts take several hours to perform, we counted the number of brain cysts on a small sample of infected mice the day before the experiment. The accuracy of brain cysts used for oral infection could be improved by pooling all infected mouse brains for cyst counts, but pooling all mouse brains and counting prior to oral infection could sacrifice parasite viability for accuracy. These are inherent limitations to studying the natural route of oral T. gondii infection.

The difference in survival between RIPK3^−/−^ and MLKL^−/−^ mice could be attributed to their ability to initiate pyroptosis, because RIPK3 can activate pyroptosis in an MLKL-dependent or -independent manner (60, 61). This result agrees with previous findings that show inhibition of pyroptosis effectors IL-1β and IL-18 improves host survival of oral T. gondii infection (17, 62–64). However, genetic knockouts in other inflammasome and pyroptosis components have shown conflicting results in host susceptibility from i.p. T. gondii infection (65–67). Our study provides further evidence that inflammasome activation in oral T. gondii infection affects the host immune response.

Our CRISPR-Cas9 ZBP1^−/−^ mouse had no significant effect on host survival of i.p. infection and reduced survival against oral challenge. This result corresponded with our previous findings, where the ZBP1^−/−^ mice also showed no significant difference to i.p. infection but reduced survival of oral infection (23). Although independent groups have linked ZBP1 function to necroptosis, they have reported opposing susceptibility phenotypes to viral infection with the previous ZBP1^−/−^ mice (25, 32). Thus, the relevance of ZBP1 to host vulnerability in infectious diseases could be remedied by this new CRISPR-Cas9 ZBP1^−/−^ mouse.

## MATERIALS AND METHODS

### Ethics statement.

All animal use was approved by and in agreement with the Institutional Animal Care and Use Committee (IACUC) at the University of Wisconsin-Madison (UW-Madison) (protocol number M005217). Cats were treated in compliance with the guidelines set by the IACUC of the U.S. Department of Agriculture, Beltsville Area (protocol number 15-017). Both institutions adhere to the regulations and guidelines set by the National Research Council. All mice were monitored daily for clinical signs of disease and were euthanized if symptoms were severe.

### Mouse experiments.

All mice used were from the C57BL/6 background. Wild-type (WT) mice were originally purchased from JAX but have been bred in the UW-Madison vivarium with all the other strains used for these studies. RIPK3 null (RIPK3^−/−^) mice were provided from Genentech (68). MLKL null (MLKL^−/−^) mice were provided by Doug Green at St. Jude Children’s Research Hospital (69). The RIPK3^−/−^ strain was genotyped by PCR with three primers: 5′-AGAAGATGCAGCAGCCTCAGCT, 5′-ACGGACCCAGGCTGACTTATCTC, and 5′-GGCACGTGCACAGGAAATAGC. The MLKL^−/−^ strain was also genotyped by PCR with three primers: 5′-TATGACCATGGCAACTCACG, 5′-ACCATCTCCCCAAACTGTGA, and 5′-TCCTTCCAGCACCTCGTAAT.

### Z-DNA binding protein-1 CRISPR-Cas9-generated mouse knockout.

The ZBP1 null (ZBP1^−/−^) mouse was generated at the UW-Madison Biotechnology Center. Two guide RNAs were designed to target the 5′ end (CGATCCCCTCTTACGTAATA; Chr:2, 173219317 to 173219336) and 3′ end (TCAATCAATCGATCAACCGC; Chr:2, 173207132 to 173207151) of *Zbp1*, removing 14 kb of genomic DNA (gDNA) that included the promoter and all splice variants (see Fig. S1A in the supplemental material). The RNA guides were optimized for the least off-target effects within the Zhang Laboratory CRISPR design tool (www.crispr.mit.edu). The guides were cloned into the plasmid pX330 or pX548 (obtained from the Zhang Laboratory via Addgene, Cambridge, MA), and the PCR products were transcribed with the T7 MEGAshortscript kit (Life Technologies). A mixture of 50 ng/μl guide RNAs and 40 ng/μl Cas9 protein in injection buffer (5 mM Trizma base, 5 mM Tris-HCl, 0.1 mM EDTA, pH 7.4) was injected into the pronucleus of 1-day-old fertilized embryos isolated from C57BL/6 mice. The resulting pups were genotyped by PCR with a mixture of three *Zbp1* primers (5′-CAACGACTGCTGCTGTCTTGC, 5′-GAACTCTGTGAAAGCCCTGTGAGG, and 5′-CCTCATCCCCTGGTTGGTGTTAC). Mice with a WT genotype produce a 376-bp band, and the ZBP1^−/−^ genotype produces a 538-bp band (Fig. S1B).

### Necroptosis assay.

Lactate dehydrogenase (LDH) release was used as a measure of necroptosis in bone marrow-derived macrophages (BMDM) from 2 independent experiments according to the manufacturer’s instructions (MAK066-1KT; Sigma-Aldrich). BMDM were harvested from 8- to 10-week-old mice and grown in 20% L929 conditioned RPMI medium as described previously (70). Harvested BMDM were seeded in technical triplicate at 5 × 10^4^ cells/well in a 96-well plate. Cells were infected with 2.5 × 10^5^ ME49 tachyzoites or left uninfected for 3 h. After infection, the cells were washed with phosphate-buffered saline (PBS; calcium and magnesium free) and stimulated with 50 ng/ml TNF (PMC3013) and 40 μM Z-VAD-FMK (ab120382) for 24 h or left unstimulated. Necrostatin-1 (SC-200142) was added at 50 μM as a control to inhibit necroptosis. Medium supernatant was used to measure LDH release at 450 nm every 5 min for 1 h in a BioTek Synergy HT plate reader. An NADH standard curve was used to determine the amount of LDH activity for each sample by the colorimetric assay.

### Parasites for *in vitro* and *in vivo* experiments.

The ME49 T. gondii strain was used for all experiments and maintained in human foreskin fibroblast cells. The mCherry parasites were generated as follows. ME49 T. gondii strain (1 × 10^7^) was electroporated with 25 μg tub-mCherry (71), linearized with KpnI, and selected with chloramphenicol. Clones were isolated by limiting dilution. Tachyzoites were injected into mice for at least 28 days, bradyzoites were collected from the brains for passage through a feline, and mCherry oocysts were collected from the feces. Oocyst concentration was calculated using a hemocytometer. Brain tissue cysts were collected from chronically infected C57BL/6J mice, and the number of brain tissue cysts for oral infection was determined by immunofluorescence as described previously (23).

### Survival curve.

Male or female mice between 9 and 13 weeks old were used for oral and intraperitoneal (i.p.) challenges. Oral challenges were performed by gavage with mCherry oocysts or bradyzoite brain cysts. The mCherry oocyst oral challenge was done with 6 × 10^3^ oocysts/mouse, and the brain tissue cyst oral challenge was done with 2 × 10^3^ or 4 × 10^3^ brain tissue cysts/mouse. The i.p. infection was performed with 1 × 10^4^ tachyzoites/mouse. All mice were monitored daily for clinical signs of disease and euthanized when moribund.

### *In vivo* parasite quantification.

Parasite burden was determined in intestine samples by mCherry fluorescence and qPCR. The UW-Madison Small Animal Imaging and Radiotherapy Facility provided an *in vivo* imaging system (IVIS) to measure mCherry fluorescence. Male and female mice at 7 to 11 weeks old were gavage fed 1 × 10^4^ mCherry oocysts/mouse in 2 independent experiments. At 7 days postinfection, the mice were sacrificed, their intestines were removed, and mCherry fluorescence was measured (excitation at 587 nm and emission at 610 nm) by IVIS. The fluorescence of infected WT and RIPK3^−/−^ mice was normalized to the average fluorescence in uninfected male and female mice. Parasite burden measured by qPCR was performed with parasite-specific SAG1 primers (72) (5′-TGCCCAGCGGGTACTACAAG and 5′-TGCCGTGTCGAGACTAGCAG) or B1 primers (73) (5′-TGCATAGGTTGCAGTCACTG and 5′-GGCGACCAATCTGCGAATACACC) on total gDNA extracted from 1-cm sections from the distal ileum in female mice orally infected with 3 × 10^4^ mCherry oocysts at 7 days postinfection by TRIzol by following the manufacturer’s instructions. A gDNA T. gondii standard curve was generated from a known concentration of parasites cultured in human foreskin fibroblast cells to calculate burden. A StepOne real-time PCR machine with iTaq Universal SYBR green supermix (1725120; Bio Rad) was used to determine parasite number in each intestine section relative to a standard curve of parasite gDNA.

### Brain cyst quantification.

The number of brain cysts was determined by immunofluorescence at 28 days after oral infection with 3 × 10^3^ mCherry oocysts in male mice. Each brain was homogenized separately with a Dounce homogenizer, fixed with 4% paraformaldehyde for 20 min, quenched with 0.1 M glycine for 2 min, and blocked for 1 h at room temperature in blocking buffer (PBS with 3% bovine serum albumin [BSA] and 0.2% Triton X-100). Brain cysts were stained with streptavidin-conjugated Dolichos biflorus agglutinin (B-1035-5; Vector Laboratories) for 1 h at room temperature, washed in PBS with 0.1% Triton X-100, and incubated with a biotinylated Alexa Flour 594 (S11227; Thermo Fisher) secondary antibody for 1 h at room temperature in the dark. An aliquot of processed brains was mounted on glass coverslips and blinded, and the number of cysts was counted on a Zeiss Axioplan III motorized microscope with a 10× objective.

### *In vitro* parasite quantification.

The parasite burden *in vitro* was determined by immunofluorescence in BMDM. A total of 1 × 10^5^ viable BMDM were seeded on glass coverslips and infected with 1 × 10^5^ mCherry tachyzoites. After 3 h postinfection, cells were either left naive or stimulated with 100 ng/ml LPS and 100 U/ml IFN-γ. At 24 and 48 h postinfection, parasites per vacuole were counted. Each time point was performed on triplicate coverslips and the experiment was repeated, providing a total of 6 coverslips per condition. mCherry fluorescence was preserved using Vectashield antifade mounting medium with 4′,6-diamidino-2-phenylindole (DAPI) (H1500; Vector Laboratories) and by photographing 10 random fields per coverslip with a Zeiss Axioplan III motorized microscope with a 40× oil objective. All photographs were blinded before counting. All parasites per vacuole were counted for every vacuole in the photograph, with at least 260 vacuoles counted per condition.

### Intestine permeability assay.

Intestine permeability was determined by LPS and FITC-dextran concentrations in mouse blood serum after oral infection. Serum LPS concentration was measured with the Pierce chromogenic endotoxin quant kit (A39552; Thermo Scientific) by following the manufacturer’s protocol. Male and female mice were orally infected with 1 × 10^4^ mCherry oocysts by gavage. As a positive control, male and female mice were treated with 3% dextran sodium sulfate (DSS) in drinking water. Paired blood serum was collected at days 0 (uninfected), 3, 5, and 7 postinfection. Blood serum LPS concentration was measured spectrophotometrically at 405 nm in a 96-well BioTek Synergy HT plate reader. Intestine permeability was determined by FITC-dextran concentration in mouse blood serum after oral infection. Female mice were infected for 7 days with 600 mCherry oocysts by gavage. Mice were fasted overnight and gavage fed 0.44 mg/g FITC-dextran, and, after 4 h, blood serum was collected at euthanasia. FITC-dextran was quantified spectrophotometrically (485-nm excitation and 528-nm emission) from blood serum.

### Intestine pathology.

Intestine pathology was evaluated by histology and length. The Comparative Pathology Laboratory at UW-Madison scored blinded hematoxylin and eosin (H&E)-stained ileum Swiss rolls from female mice infected with 600 mCherry oocysts by oral gavage at day 7 postinfection. The ileum (distal 1/3 of the small intestine) was washed with PBS and fixed in 10% buffered formalin in a Swiss roll. Uninfected female mice ileum samples were processed as controls. The slides were blinded and scored from 0 to 5 (0, equivalent to control; 5, severe) on inflammation, gland loss, smooth muscle vacuolation, and edema. The intestine length was measured in male and female mice at 7 to 10 weeks old that were gavage fed 1 × 10^4^ mCherry oocysts. The small intestine was measured at 7 days postinfection. Uninfected male and female mice 7 to 10 weeks old were used to normalize the intestine length of infected mice.

### Ileum immunohistochemistry.

Paraffin-embedded ileum sections were washed with xylene 3 times for 5 min to remove the paraffin, twice for 1 min with 100% ethanol, twice with 95% ethanol in water, once with 75% ethanol in water, and finally in water. The epitopes were retrieved by exposure to the low-pressure setting in a pressure cooker for 6 min and then washed in PBS and blocked with 3%, mass/vol, BSA in PBS at room temperature for 1 h. Primary antibody was incubated at 4°C overnight in 0.2%, vol/vol, Triton X-100 and 3%, mass/vol, BSA in PBS (1:100 Ly-6G/Ly-6C monoclonal antibody [RB6-8C5], Alexa Fluor 488 [53-5931-82; Thermo Fischer], 1:100 mCherry Monoclonal Antibody [16D7], Alexa Fluor 594 [M11240; Thermo Fischer]), washed 3 times with 3%, mass/vol, BSA in PBS, and mounted in Vectashield antifade mounting medium (VectorLabs). Samples were imaged on a Zeiss Axioplan III equipped with a triple-pass (DAPI/FITC/Texas Red) emission cube, differential interference contrast, and a monochromatic AxioCam camera operated by Zen software (Zeiss).

### Blood serum cytokine measurement.

The BD cytometric bead array mouse inflammation kit (552364; BD Biosciences) was used to measure IL-6, IL-10, monocyte chemoattractant protein-1 (MCP-1), IFN-γ, TNF, and IL-12 from blood serum. Female mice at 8 to 13 weeks old were gavage fed 6 × 10^3^ mCherry oocysts and euthanized at 7, 8, and 9 days postinfection in 3 independent experiments. Blood serum was collected for cytokine analysis by flow cytometry with a ThermoFischer Attune at the UW-Madison Flow Cytometry Core.

## Supplementary Material

Supplemental file 1
