# Supplementary material for: RIPK3 Facilitates Host Resistance to Oral Toxoplasma gondii Infection
Source: Infect Immun. 2021 Apr 16;89(5):e00021-21. doi: 10.1128/IAI.00021-21 (PMC8091083; doi:10.1128/IAI.00021-21)
Supplement: Supplemental file 1 [file IAI.00021-21-s0001.pdf]

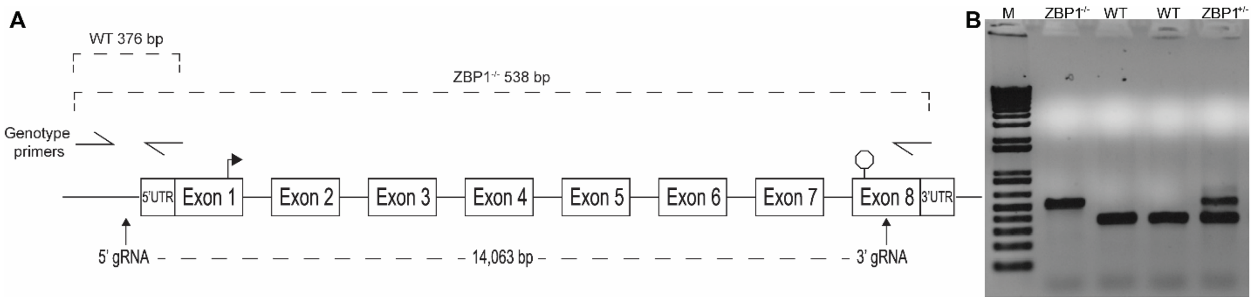

FIG S1. Generation of CRISPR-Cas9 ZBP1$^{-/-}$ mice.

(A) Two guide RNAs were designed to target the 5'-end (CGATCCCCTCTTACGTAATA; Chr:2 173219317-173219336) and 3'-end (TCAATCAATCGATCAACCGC; Chr:2 173207132-173207151) of *Zbp1*, removing 14KB of genomic DNA that included the promoter and all splice variants. Relative genotype primer locations are shown and the expected band sizes for WT (376 bp) and ZBP1$^{-/-}$ (538 bp) genotypes after PCR. (B) Genotype PCR of WT and ZBP1$^{-/-}$ mice. Lane M is a 1 Kb reference ladder. The ZBP1$^{-/-}$ lane shows a single 538 bp band and the WT lanes show a single 376 bp band for homozygous null and WT genotypes, respectively. The ZBP1$^{+/-}$ lane shows two bands, 538 bp and 376 bp, to indicate a heterozygous genotype.

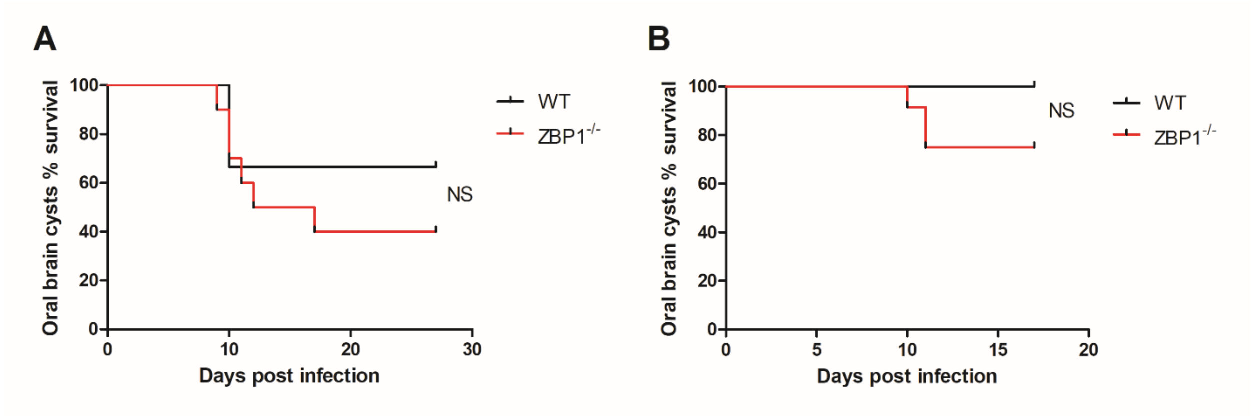

FIG S2. Independent experiments for oral brain tissue cyst survival challenges with WT and ZBP1$^{-/-}$ mice.

(A) Experiment 1. Female (WT n = 6, ZBP1$^{-/-}$ n = 10) mice were gavage fed 4 x 10^3 brain tissue cysts by gavage. (B) Experiment 2. Female (WT n = 11, ZBP1$^{-/-}$ n = 12) mice were gavage fed 2 x 10^3 brain tissue cysts by gavage. A Log-rank (Mantel-Cox) Test was performed to determine significance. Survival between WT and ZBP1$^{-/-}$ mice was not significant (NS).

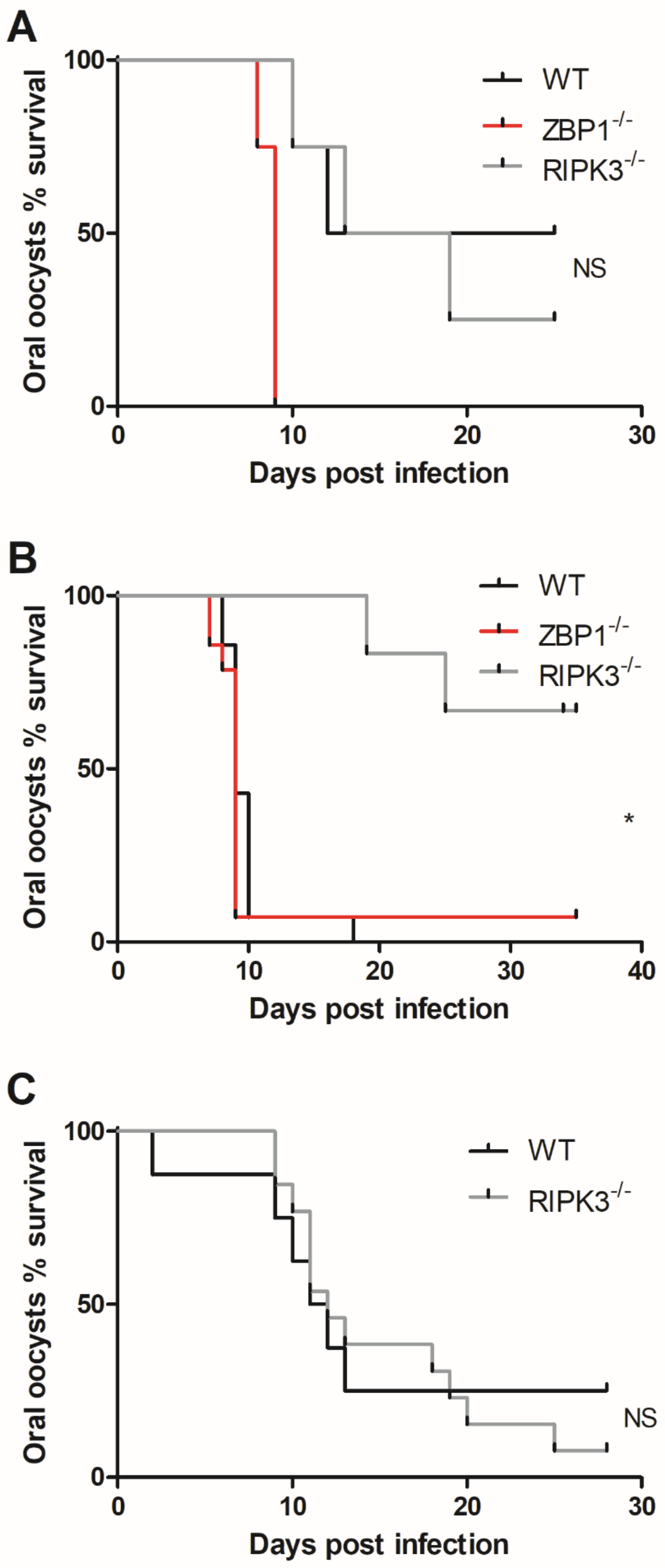
A
Oral oocysts % survival
100
50
0
0
10
20
30
Days post infection
WT
ZBP1-/-
RIPK3-/-
NS
B
Oral oocysts % survival
100
50
0
0
10
20
30
40
Days post infection
WT
ZBP1-/-
RIPK3-/-
*
C
Oral oocysts % survival
100
50
0
0
10
20
30
Days post infection
WT
RIPK3-/-
NS

FIG S3. Independent experiments for oral oocyst survival challenges.
Mice were gavage fed 6 x 10^3 mCherry oocysts. (A) Oocyst survival challenge experiment 1. Male (WT n = 4, ZBP1$^{-/-}$ n = 4, RIPK3$^{-/-}$ n = 4) mice. Survival between WT and RIPK3$^{-/-}$ mice was not significant (NS). (B) Oocyst survival challenge experiment 2. Male and female (WT n = 14, ZBP1$^{-/-}$ n = 14, RIPK3$^{-/-}$ n = 6) mice. Survival between WT and RIPK3$^{-/-}$ mice had a * P-value < 0.05. (C) Oocysts survival challenge experiment 3. Male and female (WT n = 8, RIPK3$^{-/-}$ n = 13) mice. Survival between WT and RIPK3$^{-/-}$ mice was NS. A Log-rank (Mantel-Cox) Test was performed to determine significance.

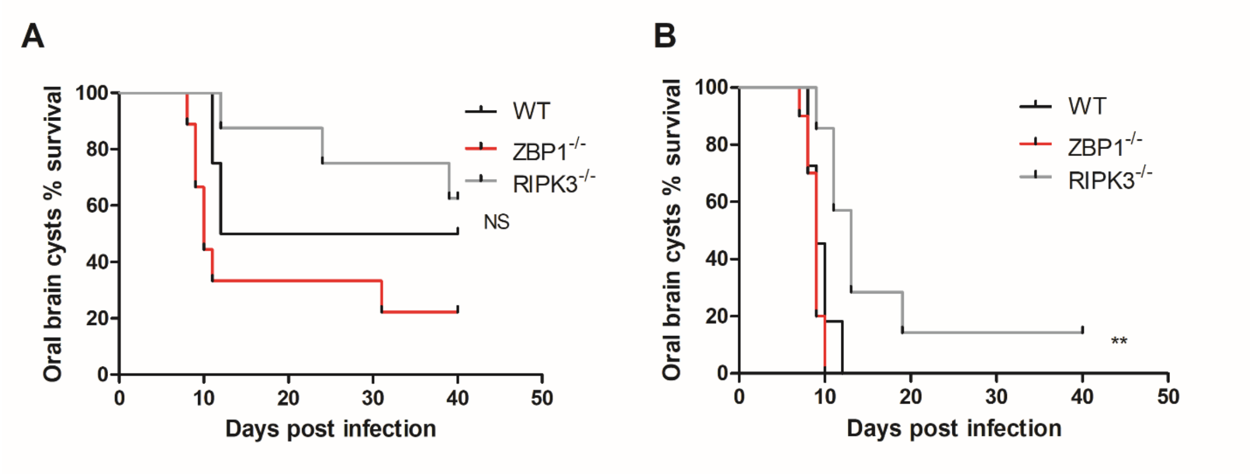

FIG S4. Independent experiments for oral brain tissue cyst survival challenges.
Mice were fed 4x10^3 brain tissue cysts. (A) Experiment 1. Female (WT n = 4, ZBP1$^{-/-}$ n = 7, RIPK3$^{-/-}$ n = 8) mice. Survival between WT and RIPK3$^{-/-}$ mice was not significant (NS). (B) Experiment 2. Female (WT n = 11, ZBP1$^{-/-}$ n = 10, RIPK3$^{-/-}$ n = 7) mice. Survival between WT and RIPK3$^{-/-}$ mice had a ** P-value < 0.01. A Log-rank (Mantel-Cox) Test was performed to determine significance.

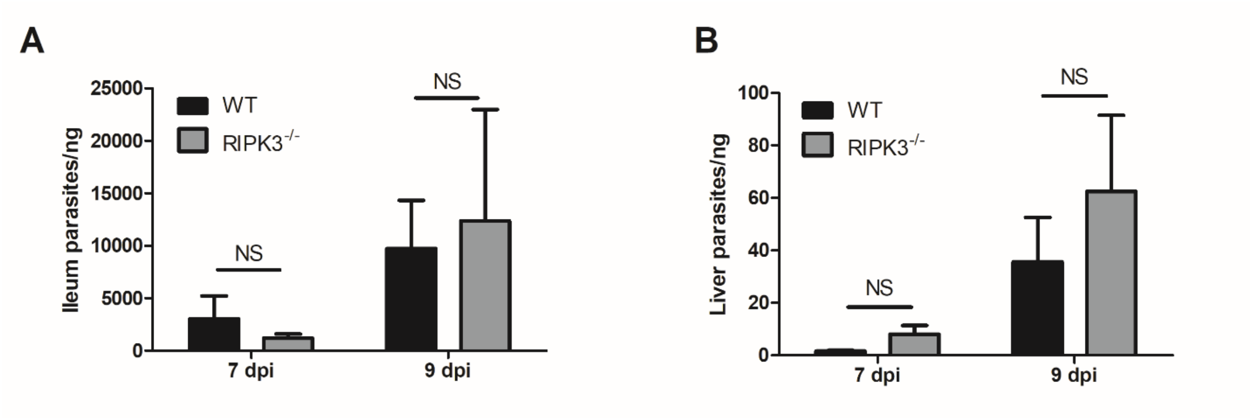

FIG S5. RIPK3$^{-/-}$ mice do not have reduced parasite burden in the intestine and liver. Female (WT n = 6, RIPK3$^{-/-}$ n = 6) mice were orally infected with 6 x 10^3 mCherry oocysts and parasite burden was measured by qPCR with parasite specific SAG1 primers at 7 and 9 dpi. (A) Intestine gDNA was extracted from 1 cm ileum tissue section. (B) Liver gDNA was extracted from 50 mg samples. A standard curve was generated from a known concentration of tachyzoite parasites to calculate burden in the ileum and liver. A 2-way ANOVA with Bonferroni post-test was used to calculate significance. Parasite burden between WT and RIPK3$^{-/-}$ mice was not significant (NS) in the ileum (A) and liver (B) at 7 or 9 dpi.

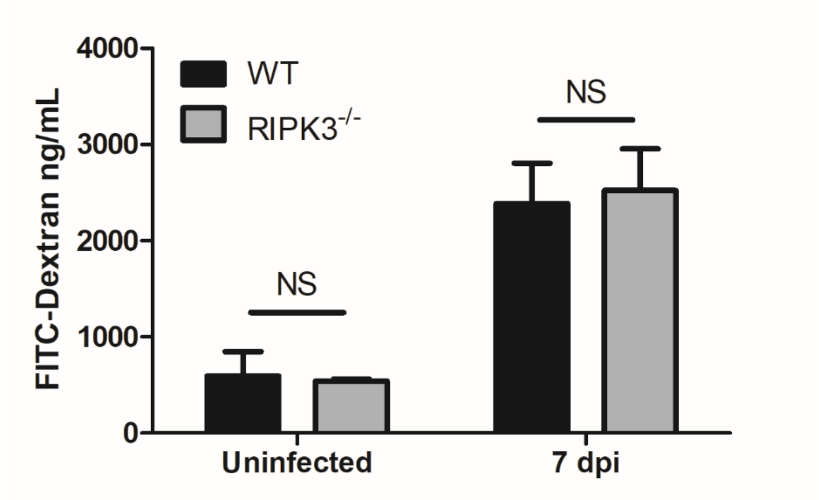

FIG S6. *T. gondii* infection creates leaky intestinal villi in both WT and RIPK3$^{-/-}$ mice. FITC-dextran concentration in blood serum from female (WT n = 9, RIPK3$^{-/-}$ n = 7) mice fed 600 mCherry oocysts by gavage. At 7 days post infection, mice were fasted overnight then gavage fed 0.44 mg/g FITC-dextran. Blood serum was collected after 4 hours and FITC-dextran was measured (485 nm excitation and 528 nm emission). A 2-way ANOVA with Bonferroni post-test was used to calculate significance. Intestine permeability was not significant (NS) between WT and RIPK3$^{-/-}$ mice at 7 dpi.

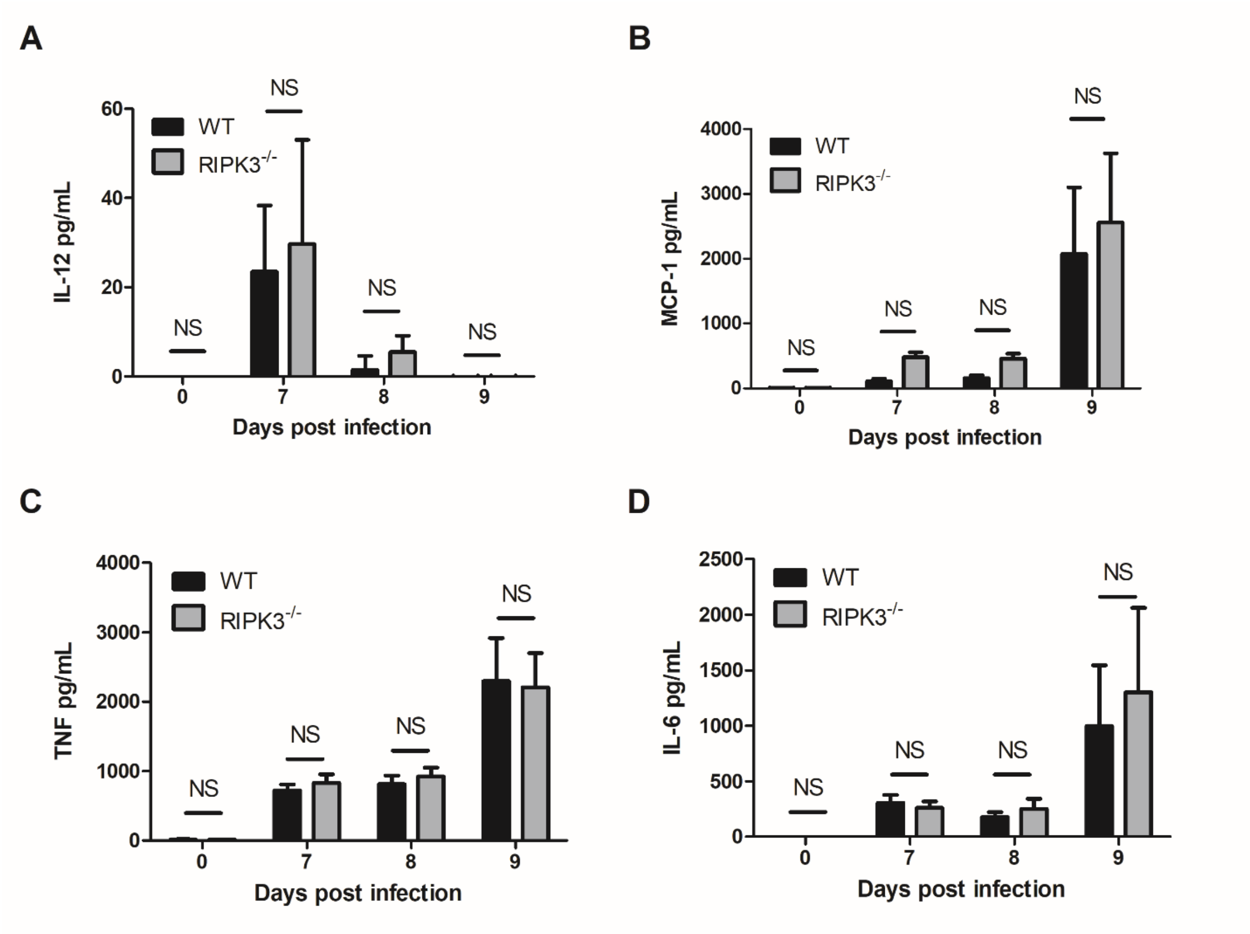

FIG S7. Inflammatory cytokines in blood serum.
Blood serum cytokines in female (WT n = 14, RIPK3-/- n = 15) mice gavage fed 6 x 10^3 mCherry oocysts. Samples were collected at 7, 8, and 9 days post infection used to measure (A) IL-12, (B) MCP-1, (C) TNF, and (D) IL-6. Each time point has at least 3 biological replicates from 3 independent experiments. A 2-way ANOVA with Bonferroni post-test was used to calculate significance. Blood serum cytokine concentration was not significant (NS) between WT and RIPK3$^{-/-}$ mice at each time point.

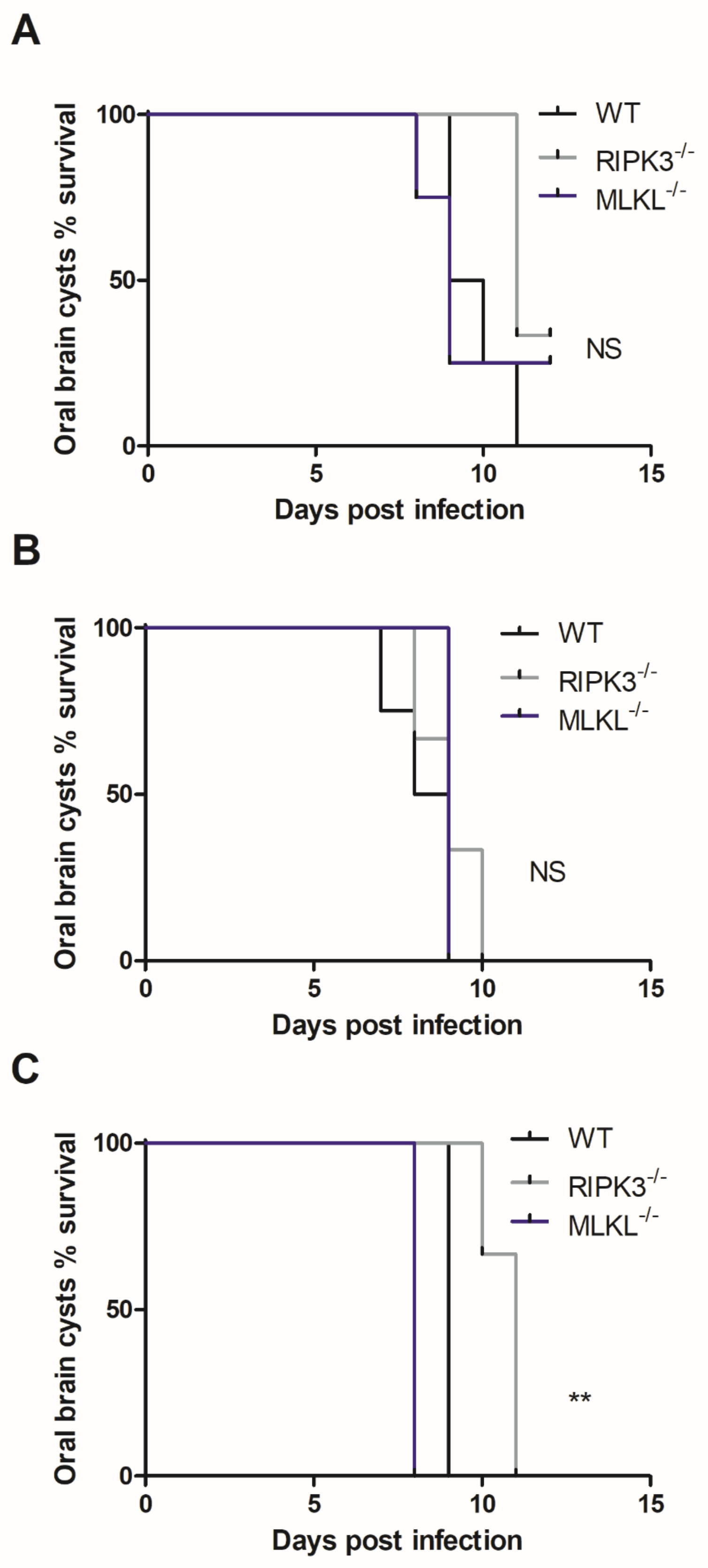
A
Oral brain cysts % survival
100
50
0
0
5
10
15
Days post infection
WT
RIPK3-/-
MLKL-/-
NS
B
Oral brain cysts % survival
100
50
0
0
5
10
15
Days post infection
WT
RIPK3-/-
MLKL-/-
NS
C
Oral brain cysts % survival
100
50
0
0
5
10
15
Days post infection
WT
RIPK3-/-
MLKL-/-
**

FIG S8. Independent MLKL$^{-/-}$ survival experiments to oral brain tissue cyst infection. (A) MLKL$^{-/-}$ brain tissue cyst survival challenge experiment 1. Female (WT n = 4, RIPK3$^{-/-}$ n = 3, MLKL$^{-/-}$ n = 4) mice. Survival was not significant (NS) between WT and RIPK3$^{-/-}$ or MLKL$^{-/-}$ mice. (B) MLKL$^{-/-}$ brain tissue cysts survival challenge experiment 2. Female (WT n = 3, RIPK3$^{-/-}$ n = 3, MLKL$^{-/-}$ n = 6) mice. Survival was NS between WT and RIPK$^{-/-}$ or MLKL$^{-/-}$ mice. (C) MLKL$^{-/-}$ brain tissue cyst survival challenge experiment 3. Female (WT n = 6, RIPK3$^{-/-}$ n = 3, MLKL$^{-/-}$ n = 6) mice. Survival between WT and RIPK3$^{-/-}$ mice had ** P-value < 0.001. Survival between WT and MLKL$^{-/-}$ had *** P-value < 0.0001. A Log-rank (Mantel-Cox) Test was performed to determine significance.
